# Supplementary material for: Optimising 7T‐fMRI for Imaging Regions of Magnetic Susceptibility
Source: Hum Brain Mapp. 2026 Aug 10;47(11):e70621. doi: 10.1002/hbm.70621 (PMC13456059; doi:10.1002/hbm.70621)
Supplement: Supplementary file 1 [file HBM-47-e70621-s001.docx]

**Supplementary Material 1: Additional tables and figures**

|  | LTP | LvATL | RITG | LFP | LmMTG | LpMTG | LIFGpt |
| --- | --- | --- | --- | --- | --- | --- | --- |
| Activation magnitude | | | | | | | |
| pTx > SESB | - | 0.2488 | 0.0240* | - | - | 0.0042** | 0.4114 |
| SESB > pTx | - | 0.7512 | 0.9760 | - | - | 0.9958 | 0.5886 |
| ME > SE | 0.0502 | 0.0001** | 0.0333* | 0.2951 | 0.0092* | 0.0003** | 0.4155 |
| SE > ME | 0.9498 | 0.9999 | 0.9667 | 0.7049 | 0.9908 | 0.9997 | 0.5845 |
| MB > SB | 0.4565 | 0.1448 | 0.1124 | 0.3495 | 0.2929 | 0.3023 | 0.3795 |
| SB > MB | 0.5435 | 0.8552 | 0.8876 | 0.6505 | 0.7071 | 0.6977 | 0.6205 |
| Interaction  (ME and MB) | 0.8990 | 0.7861 | 0.9897 | 0.7884 | 0.4673 | 0.0569 | 0.9101 |
| MEdn > ME | 0.9609 | 0.9792 | 0.9443 | 0.0783 | 0.5108 | 0.6483 | 0.7065 |
| ME > MEdn | 0.0391* | 0.0208* | 0.0557 | 0.9217 | 0.4892 | 0.3517 | 0.2935 |
| Interaction  (MEdn and ME) | 0.2570 | 0.4409 | 0.3560 | 0.3710 | 0.7106 | 0.9530 | 0.3930 |
| MBodd > SB | 0.5085 | 0.2744 | 0.0777 | - | 0.4092 | 0.3466 | 0.4490 |
| SB > MBodd | 0.4915 | 0.7256 | 0.9223 | - | 0.5908 | 0.6534 | 0.5510 |
| Interaction  (MBodd and SB) | 0.8817 | 0.8582 | 0.9969 | - | 0.7534 | 0.1315 | 0.8875 |
| Activation precision | | | | | | | |
| pTx > SESB | - | 0.4303 | 0.0191* | - | - | 0.0053** | 0.3239 |
| SESB > pTx | - | 0.5969 | 0.9809 | - | - | 0.9947 | 0.6761 |
|  |  |  |  |  |  |  |  |
| ME > SE | 0.0320* | 0.0017** | 0.0018** | 0.4773 | 0.0027** | <0.0001** | 0.0802 |
| SE > ME | 0.9680 | 0.9983 | 0.9982 | 0.5227 | 0.9973 | 1.0000 | 0.9198 |
| MB > SB | 0.4569 | 0.0007** | 0.0019** | 0.0989 | 0.2187 | 0.0266* | 0.0016** |
| SB > MB | 0.5431 | 0.9993 | 0.9981 | 0.9011 | 0.7813 | 0.9734 | 0.9984 |
| Interaction  (ME and MB) | 0.6026 | 0.8131 | 0.9958 | 0.7969 | 0.4791 | 0.0121* | 0.6575 |
| MEdn > ME | 0.3117 | <0.0001** | <0.0001** | 0.0014** | 0.1363 | <0.0001** | <0.0001** |
| ME > MEdn | 0.6883 | 1.0000 | 1.0000 | 0.9986 | 0.8637 | 1.0000 | 1.0000 |
| Interaction  (MEdn and ME) | 0.4263 | 0.8147 | 0.9537 | 0.4887 | 0.5175 | 0.6156 | 0.5693 |
| MBodd > SB | 0.4225 | 0.1057 | 0.0481* | - | 0.5697 | 0.1879 | 0.3300 |
| SB > MBodd | 0.5775 | 0.8943 | 0.9519 | - | 0.4303 | 0.8121 | 0.6700 |
| Interaction  (MBodd and SB) | 0.7872 | 0.9387 | 1.0000 | - | 0.8476 | 0.0915 | 0.7912 |
| MVPA | | | | | | | |
| pTx > SESB | - | 0.8569 | 0.3280 | - | - | 0.1529 | 0.9179 |
| SESB > pTx | - | 0.1431 | 0.6720 | - | - | 0.8471 | 0.0821 |
| ME > SE | 0.0096* | 0.0063** | 0.1612 | 0.0655 | 0.0465* | 0.0024** | 0.2508 |
| SE > ME | 0.9904 | 0.9937 | 0.8388 | 0.9345 | 0.9535 | 0.9976 | 0.7492 |
| MB > SB | 0.2455 | 0.1417 | 0.0667 | 0.3021 | 0.3949 | 0.2693 | 0.5207 |
| SB > MB | 0.7076 | 0.8460 | 0.9351 | 0.6933 | 0.5945 | 0.6776 | 0.4744 |
| Interaction  (ME and MB) | 0.6612 | 0.6182 | 0.9791 | 0.1553 | 0.1998 | 0.1660 | 0.8216 |
| MEdn > ME | 0.0027** | <0.0001** | <0.0001** | 0.0023** | <0.0001** | 0.0012** | 0.0007** |
| ME > MEdn | 0.9973 | 1.0000 | 1.0000 | 0.9977 | 0.9999 | 0.9988 | 0.9993 |
| Interaction  (MEdn and ME) | 0.2711 | 0.8973 | 0.8733 | 0.3296 | 0.8462 | 0.9714 | 0.9560 |
| MBodd > SB | 0.2584 | 0.5430 | 0.2778 | - | 0.6197 | 0.3284 | 0.6758 |
| SB > MBodd | 0.7416 | 0.4570 | 0.7222 | - | 0.3803 | 0.6716 | 0.3242 |
| Interaction  (MBodd and SB) | 0.8113 | 0.8003 | 0.9960 | - | 0.2954 | 0.1939 | 0.8072 |

Supplementary table 1: p-values for all statistical tests within regions of interest (ROIs) based on the semantic network. * = p < 0.05, ** = p < 0.05 (Bonferroni-corrected for the number of ROIs), - = ROI does not overlap by at least one voxel with the whole-brain contrast of interest (S > C) summed over all sequences in the comparison, LTP = left temporal pole, LvATL = left ventral anterior temporal lobe, RITG = right inferior temporal gyrus, LFP = left frontal pole, LmMTG = left medial middle temporal gyrus, LpMTG = left posterior middle temporal gyrus, LIFGpt = left inferior temporal gyrus pars triangularis.

|  | Cluster extent (voxels) | z-value | x | y | z | Anatomical label |
| --- | --- | --- | --- | --- | --- | --- |
| **Activation magnitude** | | | | | | |
| Comparing pTx and SESB | | | | | | |
| SESB | 8941 | 7.55 | -29 | -42 | -13 | Left temporal fusiform cortex pos |
|  |  | 7.39 | 34 | -47 | -20 | Right temporal occipital fusiform cortex |
|  |  | 7.16 | -31 | -34 | -23 | Left temporal fusiform cortex pos |
|  |  | 6.93 | -39 | -52 | -18 | Left temporal occipital fusiform cortex |
|  |  | 6.83 | 31 | -57 | -18 | Right temporal occipital fusiform cortex |
|  |  | 6.76 | 26 | -42 | -18 | Right temporal occipital fusiform cortex |
|  |  | 6.71 | -49 | -77 | 17 | Left lateral occipital cortex sup |
|  |  | 6.57 | -41 | -74 | 4 | Left lateral occipital cortex inf |
|  | 2294 | 6.75 | -46 | 43 | -3 | Left frontal pole |
|  |  | 6.73 | -54 | 40 | -8 | Left frontal pole |
|  |  | 6.72 | -49 | 33 | 10 | Left inferior frontal gyrus p tri |
|  |  | 6.43 | -44 | 20 | 20 | Left inferior frontal gyrus p ope |
|  |  | 6.18 | -51 | 36 | 24 | Left frontal pole |
|  |  | 6.13 | -39 | 6 | 34 | Left middle frontal gyrus |
|  |  | 5.94 | -36 | 16 | 24 | Left inferior frontal gyrus p ope |
|  |  | 5.5 | -41 | 8 | 22 | Left inferior frontal gyrus p ope |
|  | 232 | 5.91 | -6 | 33 | 44 | Left superior frontal gyrus |
|  | 182 | 5.59 | -26 | 16 | 42 | Left middle frontal gyrus |
|  |  | 3.6 | -39 | 20 | 57 | Left middle frontal gyrus |
|  | 160 | 5.01 | 41 | 23 | 20 | Right middle frontal gyrus |
|  |  | 4.27 | 36 | 16 | 30 | Right middle frontal gyrus |
|  | 66 | 4.3 | 41 | 33 | -8 | Right frontal orbital cortex |
|  | 110 | 4.21 | -6 | -60 | 17 | Left precuneous cortex |
|  |  | 4.1 | -4 | -52 | 12 | Left precuneous cortex |
|  |  | 3.89 | -11 | -57 | 7 | Left precuneous cortex |
| pTx | 11682 | 7.79 | -29 | -42 | -13 | Left temporal fusiform cortex pos |
|  |  | 7.69 | 34 | -47 | -20 | Right temporal occipital fusiform cortex |
|  |  | 7.52 | 36 | -67 | -16 | Right occipital fusiform gyrus |
|  |  | 7.52 | -39 | -52 | -18 | Left temporal occipital fusiform cortex |
|  |  | 7.43 | -36 | -42 | -20 | Left temporal fusiform cortex pos |
|  |  | 7.34 | 41 | -42 | -18 | Right temporal occipital fusiform cortex |
|  |  | 7.3 | -29 | -32 | -20 | Left parahippocampal gyrus pos |
|  |  | 7.17 | 46 | -77 | -8 | Right lateral occipital cortex inf |
|  | 3242 | 6.79 | -54 | 40 | -8 | Left frontal pole |
|  |  | 6.72 | -51 | 36 | 24 | Left frontal pole |
|  |  | 6.67 | -46 | 43 | -3 | Left frontal pole |
|  |  | 6.67 | -49 | 33 | 10 | Left inferior frontal gyrus p tri |
|  |  | 6.63 | -39 | 6 | 34 | Left middle frontal gyrus |
|  |  | 6.59 | -44 | 20 | 20 | Left inferior frontal gyrus p ope |
|  |  | 6.22 | -51 | 20 | 30 | Left middle frontal gyrus |
|  |  | 5.83 | -29 | 18 | 62 | Left superior frontal gyrus |
|  | 501 | 6.1 | 14 | -74 | -30 | Right cerebellum |
|  |  | 6.06 | 9 | -82 | -30 | Right cerebellum |
|  |  | 5.34 | 34 | -74 | -48 | Right cerebellum |
|  | 296 | 5.57 | 44 | 26 | 20 | Right inferior frontal gyrus p tri |
|  |  | 4.59 | 36 | 16 | 30 | Right middle frontal gyrus |
|  |  | 3.28 | 49 | 33 | 34 | Right middle frontal gyrus |
|  | 97 | 4.61 | 24 | -60 | 20 | Right precuneous cortex |
|  |  | 4.03 | 19 | -50 | 10 | Right precuneous cortex |
|  |  | 4.01 | 11 | -52 | 14 | Right precuneous cortex |
|  | 146 | 4.4 | 34 | 38 | -13 | Right frontal pole |
|  |  | 4.2 | 31 | 33 | -20 | Right frontal pole |
|  |  | 3.63 | 34 | 30 | 0 | Right frontal orbital cortex |
|  |  | 3.53 | 51 | 33 | -16 | Right frontal orbital cortex |
|  |  | 3.43 | 46 | 26 | -8 | Right frontal orbital cortex |
| ANOVA | 126 | 4.62 | 36 | -67 | -16 | Right occipital fusiform gyrus |
|  |  | 4.08 | 39 | -77 | -10 | Right lateral occipital cortex inf |
|  |  | 3.9 | 31 | -80 | -13 | Right occipital fusiform gyrus |
|  |  | 3.39 | 51 | -72 | 0 | Right lateral occipital cortex inf |
| pTx > SESB | 172 | 4.76 | 36 | -67 | -16 | Right occipital fusiform gyrus |
|  |  | 4.24 | 39 | -77 | -10 | Right lateral occipital cortex inf |
|  |  | 4.07 | 31 | -80 | -13 | Right occipital fusiform gyrus |
|  |  | 3.57 | 51 | -72 | 0 | Right lateral occipital cortex inf |
|  |  | 3.18 | 54 | -70 | 7 | Right lateral occipital cortex inf |
| 2 x 2 factorial design – echo and band | | | | | | |
| SESB | 11459 | Inf | -31 | -44 | -20 | Left temporal fusiform cortex pos |
|  |  | Inf | 34 | -47 | -20 | Right temporal occipital fusiform cortex |
|  |  | Inf | 36 | -40 | -23 | Right temporal occipital fusiform cortex |
|  |  | Inf | -39 | -57 | -16 | Left temporal occipital fusiform cortex |
|  |  | Inf | 46 | -80 | 0 | Right lateral occipital cortex inf |
|  |  | Inf | -44 | -77 | 4 | Left lateral occipital cortex inf |
|  |  | Inf | 49 | -67 | 12 | Right lateral occipital cortex inf |
|  |  | Inf | 31 | -44 | -13 | Right temporal occipital fusiform cortex |
|  | 2394 | Inf | -49 | 33 | 10 | Left inferior frontal gyrus p tri |
|  |  | 7.68 | -39 | 10 | 24 | Left inferior frontal gyrus p ope |
|  |  | 7.25 | -46 | 20 | 22 | Left inferior frontal gyrus p ope |
|  |  | 7.2 | -46 | 43 | -3 | Left frontal pole |
|  |  | 7.12 | -49 | 33 | -3 | Left inferior frontal gyrus p tri |
|  |  | 6.84 | -44 | 40 | -10 | Left frontal pole |
|  |  | 6.66 | -56 | 18 | 27 | Left inferior frontal gyrus p ope |
|  |  | 6.62 | -59 | 23 | 14 | Left inferior frontal gyrus p tri |
|  | 304 | 7.68 | 39 | 20 | 20 | Right inferior frontal gyrus p ope |
|  |  | 4.78 | 54 | 30 | 12 | Right inferior frontal gyrus p tri |
|  | 276 | 6.44 | -6 | 23 | 50 | Left superior frontal gyrus |
|  |  | 5.12 | -6 | 33 | 44 | Left superior frontal gyrus |
|  | 206 | 5.89 | -29 | 20 | 52 | Left middle frontal gyrus |
|  |  | 4.09 | -39 | 20 | 57 | Left middle frontal gyrus |
|  |  | 3.78 | -34 | 10 | 64 | Left middle frontal gyrus |
|  | 140 | 5.4 | 34 | -70 | -46 | Right cerebellum |
|  |  | 3.49 | 24 | -77 | -43 | Right cerebellum |
|  | 109 | 4.95 | 11 | -77 | -28 | Right cerebellum |
|  |  | 4.06 | 14 | -80 | -36 | Right cerebellum |
|  | 113 | 4.4 | -9 | -52 | 14 | Left precuneous cortex |
|  |  | 4.33 | -11 | -54 | 4 | Left precuneous cortex |
| SEMB | 20421 | Inf | -31 | -44 | -20 | Left temporal fusiform cortex pos |
|  |  | Inf | 34 | -47 | -20 | Right temporal occipital fusiform cortex |
|  |  | Inf | 49 | -67 | 12 | Right lateral occipital cortex inf |
|  |  | Inf | 44 | -77 | -3 | Right lateral occipital cortex inf |
|  |  | Inf | -39 | -57 | -16 | Left temporal occipital fusiform cortex |
|  |  | Inf | 31 | -34 | -20 | Right temporal fusiform cortex pos |
|  |  | Inf | 39 | -40 | -23 | Right temporal occipital fusiform cortex |
|  |  | Inf | 36 | -64 | -16 | Right occipital fusiform gyrus |
|  | 568 | 7.7 | 39 | 20 | 17 | Right inferior frontal gyrus p ope |
|  |  | 7.46 | 49 | 28 | 17 | Right inferior frontal gyrus p tri |
|  |  | 6.37 | 39 | 16 | 30 | Right middle frontal gyrus |
|  |  | 3.95 | 19 | 10 | 32 | Right lateral ventricle |
|  | 225 | 5.46 | 44 | 30 | -6 | Right frontal orbital cortex |
|  |  | 4.57 | 34 | 33 | -18 | Right frontal pole |
|  | 203 | 5.09 | -14 | 3 | 10 | left anterior thalamic radiation |
|  |  | 5.05 | -9 | -12 | 4 | left thalamus |
| MESB | 22950 | Inf | -31 | -44 | -20 | Left temporal fusiform cortex pos |
|  |  | Inf | 34 | -47 | -20 | Right temporal occipital fusiform cortex |
|  |  | Inf | -44 | -50 | -16 | Left inferior temporal gyrus temocc |
|  |  | Inf | -39 | -57 | -18 | Left temporal occipital fusiform cortex |
|  |  | Inf | 36 | -67 | -16 | Right occipital fusiform gyrus |
|  |  | Inf | 34 | -60 | -18 | Right temporal occipital fusiform cortex |
|  |  | Inf | -41 | -40 | -18 | Left temporal fusiform cortex pos |
|  |  | Inf | -34 | -70 | -16 | Left occipital fusiform gyrus |
|  | 733 | Inf | 41 | 20 | 22 | Right inferior frontal gyrus p ope |
|  |  | Inf | 49 | 28 | 20 | Right inferior frontal gyrus p tri |
|  |  | 6.23 | 54 | 33 | 14 | Right inferior frontal gyrus p tri |
|  |  | 4.57 | 54 | 30 | 4 | Right inferior frontal gyrus p tri |
|  | 468 | 7.75 | 31 | 36 | -13 | Right frontal pole |
|  |  | 5.91 | 39 | 28 | -3 | Right frontal orbital cortex |
|  | 212 | 6.95 | -11 | -82 | -33 | Left cerebellum |
| MEMB | 20648 | Inf | -34 | -44 | -23 | Left temporal fusiform cortex pos |
|  |  | Inf | 36 | -42 | -23 | Right temporal occipital fusiform cortex |
|  |  | Inf | -36 | -57 | -16 | Left temporal occipital fusiform cortex |
|  |  | Inf | -44 | -50 | -16 | Left inferior temporal gyrus temocc |
|  |  | Inf | 34 | -60 | -18 | Right temporal occipital fusiform cortex |
|  |  | Inf | 36 | -67 | -16 | Right occipital fusiform gyrus |
|  |  | Inf | -34 | -70 | -16 | Left occipital fusiform gyrus |
|  |  | Inf | -44 | -40 | -18 | Left temporal fusiform cortex pos |
|  | 461 | 7.65 | 41 | 23 | 20 | Right middle frontal gyrus |
|  |  | 7.24 | 49 | 28 | 20 | Right inferior frontal gyrus p tri |
|  | 234 | 6.64 | 31 | 36 | -13 | Right frontal pole |
|  |  | 4.67 | 41 | 30 | -6 | Right frontal orbital cortex |
|  | 167 | 6.09 | -9 | -84 | -33 | Left cerebellum |
| ANOVA | 268 | 5.3 | -44 | -40 | -18 | Left temporal fusiform cortex pos |
|  |  | 4.71 | -51 | -57 | -20 | Left inferior temporal gyrus temocc |
|  |  | 4.39 | -59 | -50 | -16 | Left inferior temporal gyrus temocc |
|  |  | 4.09 | -36 | -44 | -26 | Left temporal occipital fusiform cortex |
|  |  | 3.77 | -41 | -47 | -8 | left inferior longitudinal fas |
|  |  | 3.63 | -66 | -54 | -6 | Left middle temporal gyrus temocc |
|  | 108 | 4.44 | -34 | 18 | 20 | Left inferior frontal gyrus p ope |
|  |  | 4.09 | -36 | 18 | 32 | Left middle frontal gyrus |
|  |  | 3.61 | -44 | 26 | 22 | Left inferior frontal gyrus p tri |
|  |  | 3.33 | -39 | 26 | 14 | Left inferior frontal gyrus p tri |
|  | 80 | 4.31 | 41 | -54 | 17 | Right angular gyrus |
|  |  | 4.08 | 46 | -67 | 10 | Right lateral occipital cortex inf |
|  |  | 3.64 | 29 | -60 | 0 | Right lingual gyrus |
|  |  | 3.62 | 34 | -70 | 4 | right inferior longitudinal fas |
|  | 123 | 4.27 | 26 | -47 | -3 | Right lingual gyrus |
|  |  | 4.18 | 34 | -44 | -16 | Right temporal occipital fusiform cortex |
|  |  | 3.97 | 24 | -32 | -13 | right cingulum hipp |
|  |  | 3.86 | 31 | -24 | -20 | Right parahippocampal gyrus pos |
|  |  | 3.5 | 34 | -34 | -20 | Right temporal fusiform cortex pos |
| ME > SE | 620 | 5.98 | -44 | -40 | -18 | Left temporal fusiform cortex pos |
|  |  | 5.27 | -51 | -57 | -20 | Left inferior temporal gyrus temocc |
|  |  | 5.12 | -59 | -50 | -16 | Left inferior temporal gyrus temocc |
|  |  | 4.6 | -36 | -44 | -26 | Left temporal occipital fusiform cortex |
|  |  | 4.51 | -41 | -47 | -10 | left inferior longitudinal fas |
|  |  | 4.43 | -66 | -54 | -6 | Left middle temporal gyrus temocc |
|  |  | 4.18 | -44 | -24 | -20 | Left inferior temporal gyrus post |
|  |  | 3.53 | -36 | -24 | -26 | Left temporal fusiform cortex pos |
|  | 101 | 5.44 | 34 | -40 | -28 | Right temporal fusiform cortex pos |
|  |  | 3.55 | 46 | -40 | -20 | Right inferior temporal gyrus temocc |
|  | 117 | 5.11 | -21 | 36 | -10 | Left frontal orbital cortex |
|  |  | 3.68 | -34 | 36 | -10 | Left frontal orbital cortex |
|  |  | 3.43 | -9 | 23 | -13 | Left subcallosal cortex |
| 2 x 2 factorial design – denoising and band | | | | | | |
| MEdn > ME | 323 | 4.62 | -31 | -47 | 2 | Left lateral ventricle |
|  |  | 4.22 | -14 | -34 | 4 | left thalamus |
|  |  | 4.13 | -29 | -37 | 14 | left anterior thalamic radiation |
|  |  | 4.08 | 4 | -32 | 12 | 3rd ventricle |
|  |  | 4.08 | -16 | -42 | 17 | Left lateral ventricle |
|  |  | 4.05 | 11 | -32 | 4 | right thalamus |
|  | 104 | 4.55 | -1 | -52 | -56 | Left cerebellum |
|  |  | 3.75 | -11 | -40 | -50 | brain stem |
|  |  | 3.59 | 4 | -54 | -66 | brain stem |
|  | 135 | 3.94 | 29 | -34 | 4 | Right lateral ventricle |
|  |  | 3.81 | 29 | -34 | 17 | Right lateral ventricle |
|  |  | 3.79 | 21 | -24 | 22 | right corticospinal tract |
|  |  | 3.64 | 31 | -44 | 0 | Right lateral ventricle |
| 2 x 2 factorial design – echo and (downsampled) band | | | | | | |
| ANOVA | 224 | 4.77 | -49 | -52 | -20 | Left inferior temporal gyrus temocc |
|  |  | 4.72 | -44 | -40 | -18 | Left temporal fusiform cortex pos |
|  |  | 4.02 | -41 | -47 | -8 | left inferior longitudinal fas |
|  |  | 4.02 | -59 | -50 | -16 | Left inferior temporal gyrus temocc |
|  |  | 3.68 | -39 | -44 | -26 | Left temporal fusiform cortex pos |
|  | 85 | 4.34 | 41 | -57 | 17 | Right angular gyrus |
|  |  | 4.18 | 44 | -67 | 10 | Right lateral occipital cortex inf |
|  | 89 | 4.19 | -34 | 13 | 20 | Left inferior frontal gyrus p ope |
|  |  | 4.04 | -36 | 18 | 32 | Left middle frontal gyrus |
|  |  | 3.56 | -44 | 28 | 22 | Left middle frontal gyrus |
| **Activation precision** | | | | | | |
| Comparing pTx and SESB | | | | | | |
| SESB | 9906 | 7.62 | -29 | -42 | -13 | Left temporal fusiform cortex pos |
|  |  | 7.35 | 39 | -44 | -18 | Right temporal occipital fusiform cortex |
|  |  | 7.29 | 34 | -52 | -20 | Right temporal occipital fusiform cortex |
|  |  | 7.18 | -34 | -50 | -20 | Left temporal occipital fusiform cortex |
|  |  | 7.18 | 34 | -67 | -16 | Right occipital fusiform gyrus |
|  |  | 7.06 | 24 | -42 | -18 | Right temporal occipital fusiform cortex |
|  |  | 7.01 | 36 | -60 | -18 | Right temporal occipital fusiform cortex |
|  |  | 7 | 51 | -70 | 7 | Right lateral occipital cortex inf |
|  | 2162 | 6.27 | -54 | 40 | -8 | Left frontal pole |
|  |  | 5.97 | -51 | 36 | 10 | Left inferior frontal gyrus p tri |
|  |  | 5.91 | -41 | 13 | 22 | Left inferior frontal gyrus p ope |
|  |  | 5.89 | -54 | 30 | 17 | Left inferior frontal gyrus p tri |
|  |  | 5.68 | -36 | 36 | -13 | Left frontal orbital cortex |
|  |  | 5.56 | -51 | 20 | 22 | Left inferior frontal gyrus p ope |
|  |  | 5.36 | -46 | 36 | -16 | Left frontal pole |
|  |  | 5.3 | -39 | 6 | 34 | Left middle frontal gyrus |
|  | 383 | 5.47 | -24 | 16 | 44 | Left superior frontal gyrus |
|  |  | 5.14 | -9 | 23 | 47 | Left superior frontal gyrus |
|  |  | 4.97 | -29 | 23 | 57 | Left superior frontal gyrus |
|  |  | 4.68 | -6 | 30 | 44 | Left superior frontal gyrus |
|  |  | 3.81 | -14 | 18 | 42 | Left paracingulate gyrus |
|  | 165 | 5.43 | 31 | 0 | -36 | Right temporal fusiform cortex ant |
|  |  | 3.69 | 31 | -10 | -36 | Right temporal fusiform cortex pos |
|  | 185 | 5.19 | 44 | 26 | 17 | Right inferior frontal gyrus p tri |
|  | 129 | 4.83 | 14 | -74 | -30 | Right cerebellum |
|  |  | 3.73 | 14 | -90 | -30 | Right cerebellum |
|  | 121 | 4.59 | -6 | -60 | 17 | Left precuneous cortex |
|  |  | 3.7 | -16 | -57 | 10 | Left precuneous cortex |
|  | 91 | 4.44 | 36 | -64 | -38 | Right cerebellum |
|  |  | 3.9 | 34 | -67 | -48 | Right cerebellum |
|  |  | 3.8 | 29 | -72 | -40 | Right cerebellum |
| pTx | 12474 | 7.73 | 34 | -67 | -16 | Right occipital fusiform gyrus |
|  |  | 7.7 | 34 | -52 | -20 | Right temporal occipital fusiform cortex |
|  |  | 7.7 | -29 | -42 | -13 | Left temporal fusiform cortex pos |
|  |  | 7.68 | 39 | -44 | -18 | Right temporal occipital fusiform cortex |
|  |  | 7.53 | 24 | -42 | -18 | Right temporal occipital fusiform cortex |
|  |  | 7.32 | 36 | -60 | -18 | Right temporal occipital fusiform cortex |
|  |  | 7.26 | -36 | -50 | -20 | Left temporal occipital fusiform cortex |
|  |  | 7.21 | 34 | -37 | -20 | Right temporal fusiform cortex pos |
|  | 2923 | 6.34 | -54 | 40 | -8 | Left frontal pole |
|  |  | 6.22 | -54 | 30 | 17 | Left inferior frontal gyrus p tri |
|  |  | 6 | -41 | 16 | 22 | Left inferior frontal gyrus p ope |
|  |  | 5.97 | -39 | 6 | 34 | Left middle frontal gyrus |
|  |  | 5.95 | -51 | 40 | 7 | Left frontal pole |
|  |  | 5.92 | -46 | 43 | -3 | Left frontal pole |
|  |  | 5.86 | -46 | 36 | -16 | Left frontal pole |
|  |  | 5.81 | -51 | 38 | 24 | Left frontal pole |
|  | 333 | 5.82 | 44 | 26 | 17 | Right inferior frontal gyrus p tri |
|  |  | 4.17 | 36 | 16 | 30 | Right middle frontal gyrus |
|  | 637 | 5.74 | 9 | -84 | -33 | Right cerebellum |
|  |  | 5.44 | 14 | -77 | -30 | Right cerebellum |
|  |  | 5.26 | 34 | -74 | -53 | Right cerebellum |
|  |  | 4.69 | 36 | -70 | -38 | Right cerebellum |
|  |  | 4.26 | 24 | -82 | -48 | Right cerebellum |
|  | 336 | 5.21 | -6 | 30 | 44 | Left superior frontal gyrus |
|  |  | 4.4 | -6 | 43 | 30 | Left paracingulate gyrus |
|  |  | 4.36 | -1 | 28 | 52 | Left superior frontal gyrus |
|  |  | 3.47 | -11 | 53 | 37 | Left frontal pole |
|  | 179 | 4.7 | 24 | -60 | 17 | Right supracalcarine cortex |
|  |  | 4.41 | 21 | -57 | 10 | Right precuneous cortex |
|  |  | 4.27 | 9 | -52 | 10 | Right precuneous cortex |
|  | 159 | 4.43 | 34 | 38 | -16 | Right frontal pole |
|  |  | 4.11 | 51 | 33 | -16 | Right frontal orbital cortex |
|  |  | 4.06 | 34 | 30 | 0 | Right frontal orbital cortex |
|  |  | 3.4 | 44 | 38 | -23 | Right frontal pole |
| 2 x 2 factorial design – echo and band | | | | | | |
| SESB | 9047 | Inf | -29 | -44 | -18 | Left temporal occipital fusiform cortex |
|  |  | Inf | 36 | -42 | -20 | Right temporal occipital fusiform cortex |
|  |  | Inf | -29 | -52 | -10 | Left temporal occipital fusiform cortex |
|  |  | Inf | 49 | -67 | 12 | Right lateral occipital cortex inf |
|  |  | Inf | 31 | -60 | -16 | Right temporal occipital fusiform cortex |
|  |  | 7.7 | 26 | -50 | -13 | Right temporal occipital fusiform cortex |
|  |  | 7.69 | -46 | -74 | 12 | Left lateral occipital cortex inf |
|  |  | 7.68 | 34 | -34 | -23 | Right temporal fusiform cortex pos |
|  | 1738 | 6.46 | -49 | 33 | 7 | Left inferior frontal gyrus p tri |
|  |  | 6.37 | -39 | 13 | 22 | Left inferior frontal gyrus p ope |
|  |  | 6.19 | -44 | 26 | 12 | Left inferior frontal gyrus p tri |
|  |  | 6.18 | -46 | 20 | 20 | Left inferior frontal gyrus p ope |
|  |  | 5.13 | -41 | 40 | -10 | Left frontal pole |
|  |  | 5.12 | -44 | 33 | -13 | Left frontal orbital cortex |
|  |  | 5.11 | -46 | 46 | 0 | Left frontal pole |
|  |  | 4.85 | -49 | 38 | -18 | Left frontal pole |
|  | 213 | 6.28 | 36 | 20 | 20 | Right inferior frontal gyrus p ope |
|  |  | 5.53 | 49 | 28 | 17 | Right inferior frontal gyrus p tri |
|  | 136 | 5.16 | -6 | 23 | 47 | Left superior frontal gyrus |
|  |  | 4.04 | -9 | 33 | 44 | Left superior frontal gyrus |
|  | 123 | 4.62 | -29 | 20 | 54 | Left middle frontal gyrus |
| SEMB | 22882 | Inf | -29 | -47 | -18 | Left temporal occipital fusiform cortex |
|  |  | Inf | 46 | -67 | 12 | Right lateral occipital cortex inf |
|  |  | Inf | 34 | -34 | -23 | Right temporal fusiform cortex pos |
|  |  | Inf | -46 | -77 | 10 | Left lateral occipital cortex inf |
|  |  | Inf | 29 | -54 | -13 | Right temporal occipital fusiform cortex |
|  |  | Inf | 36 | -42 | -20 | Right temporal occipital fusiform cortex |
|  |  | Inf | -26 | -52 | -10 | Left temporal occipital fusiform cortex |
|  |  | Inf | -41 | -80 | -10 | Left lateral occipital cortex inf |
|  | 452 | Inf | 51 | 28 | 17 | Right inferior frontal gyrus p tri |
|  |  | 4.7 | 39 | 16 | 30 | Right middle frontal gyrus |
|  | 248 | 5.42 | 34 | 33 | -20 | Right frontal pole |
|  |  | 5.22 | 44 | 33 | -8 | Right frontal pole |
|  | 104 | 5.36 | 6 | -62 | -50 | Right cerebellum |
|  |  | 4.9 | 9 | -54 | -43 | Right cerebellum |
|  | 198 | 5.35 | -6 | 20 | -28 | Left subcallosal cortex |
|  |  | 3.55 | -4 | 43 | -30 | Left frontal medial cortex |
|  |  | 3.37 | -11 | 10 | -26 | Left frontal orbital cortex |
|  |  | 3.32 | 6 | 23 | -26 | Right subcallosal cortex |
|  | 210 | 5.35 | -9 | -12 | 7 | left thalamus |
|  |  | 5.03 | -14 | 3 | 4 | left anterior thalamic radiation |
|  | 141 | 5.16 | -9 | -82 | -33 | Right cerebellum |
|  | 104 | 5.1 | -9 | 63 | -18 | Left frontal pole |
| MESB | 23065 | Inf | -31 | -44 | -18 | Left temporal occipital fusiform cortex |
|  |  | Inf | 36 | -42 | -20 | Right temporal occipital fusiform cortex |
|  |  | Inf | -39 | -42 | -16 | Left temporal fusiform cortex pos |
|  |  | Inf | -46 | -74 | 10 | Left lateral occipital cortex inf |
|  |  | Inf | 34 | -34 | -23 | Right temporal fusiform cortex pos |
|  |  | Inf | -26 | -52 | -13 | Left temporal occipital fusiform cortex |
|  |  | Inf | 34 | -54 | -18 | Right temporal occipital fusiform cortex |
|  |  | Inf | -44 | -50 | -13 | Left inferior temporal gyrus temocc |
|  | 1169 | 7.74 | 36 | 20 | 20 | Right inferior frontal gyrus p ope |
|  |  | 7.72 | 49 | 28 | 17 | Right inferior frontal gyrus p tri |
|  |  | 6.92 | 34 | 36 | -13 | Right frontal pole |
|  |  | 6.03 | 31 | 10 | 30 | Right middle frontal gyrus |
|  |  | 5.87 | 41 | 28 | -3 | Right frontal orbital cortex |
|  | 216 | 5.84 | -9 | -82 | -33 | Left cerebellum |
|  | 130 | 5.24 | 6 | -54 | -43 | Right cerebellum |
|  |  | 5.01 | 4 | -62 | -50 | Right cerebellum |
|  |  | 3.45 | -6 | -60 | -48 | Left cerebellum |
| MEMB | 23862 | Inf | -31 | -44 | -18 | Left temporal occipital fusiform cortex |
|  |  | Inf | -36 | -52 | -18 | Left temporal occipital fusiform cortex |
|  |  | Inf | -44 | -77 | 2 | Left lateral occipital cortex inf |
|  |  | Inf | -49 | -74 | 10 | Left lateral occipital cortex inf |
|  |  | Inf | 34 | -44 | -20 | Right temporal occipital fusiform cortex |
|  |  | Inf | -41 | -42 | -18 | Left temporal fusiform cortex pos |
|  |  | Inf | -31 | -64 | -13 | Left occipital fusiform gyrus |
|  |  | Inf | -44 | -50 | -13 | Left inferior temporal gyrus temocc |
|  | 1016 | Inf | 49 | 28 | 17 | Right inferior frontal gyrus p tri |
|  |  | 7.76 | 39 | 23 | 17 | Right inferior frontal gyrus p tri |
|  |  | 6.83 | 34 | 36 | -13 | Right frontal pole |
|  |  | 6.42 | 36 | 13 | 32 | Right middle frontal gyrus |
|  |  | 5.55 | 41 | 28 | -3 | Right frontal orbital cortex |
|  |  | 4.66 | 49 | 33 | 7 | Right frontal pole |
|  | 223 | 6.3 | -9 | -84 | -36 | Left cerebellum |
|  | 143 | 5.46 | 4 | -57 | -48 | Right cerebellum |
|  |  | 5.3 | 6 | -54 | -40 | Right cerebellum |
|  |  | 3.66 | -6 | -57 | -48 | Left cerebellum |
|  | 90 | 5.28 | -21 | 68 | 17 | Left frontal pole |
| ANOVA | 2729 | 6.34 | -34 | -44 | -20 | Left temporal fusiform cortex pos |
|  |  | 6.15 | -44 | -40 | -18 | Left temporal fusiform cortex pos |
|  |  | 6.14 | -44 | -77 | 2 | Left lateral occipital cortex inf |
|  |  | 6.07 | -49 | -77 | 10 | Left lateral occipital cortex inf |
|  |  | 5.99 | -44 | -50 | -16 | Left inferior temporal gyrus temocc |
|  |  | 5.88 | -36 | -72 | -16 | Left occipital fusiform gyrus |
|  |  | 5.69 | -41 | -80 | -10 | Left lateral occipital cortex inf |
|  |  | 5.35 | -51 | -44 | -13 | left superior longitudinal fas |
|  | 2159 | 5.73 | 46 | -67 | 12 | Right lateral occipital cortex inf |
|  |  | 5.64 | 36 | -40 | -28 | Right temporal fusiform cortex pos |
|  |  | 5.6 | 36 | -74 | -13 | Right occipital fusiform gyrus |
|  |  | 5.44 | 46 | -77 | -3 | Right lateral occipital cortex inf |
|  |  | 5.19 | 29 | -60 | -13 | Right temporal occipital fusiform cortex |
|  |  | 5.17 | 44 | -32 | -23 | Right inferior temporal gyrus post |
|  |  | 5.05 | 46 | -77 | 7 | Right lateral occipital cortex inf |
|  |  | 4.96 | 36 | -52 | -20 | Right temporal occipital fusiform cortex |
|  | 188 | 5.01 | -21 | 36 | -10 | Left frontal orbital cortex |
|  |  | 4.06 | 6 | 6 | -16 | Right subcallosal cortex |
|  |  | 3.84 | 4 | 13 | -13 | Right subcallosal cortex |
|  |  | 3.81 | -9 | 26 | -13 | Left subcallosal cortex |
|  |  | 3.5 | -34 | 33 | -13 | Left frontal orbital cortex |
|  | 90 | 5 | 34 | 3 | -38 | Right temporal pole |
|  |  | 4.13 | 34 | -12 | -30 | Right parahippocampal gyrus ant |
|  |  | 4.12 | 34 | 8 | -48 | Right temporal pole |
|  | 501 | 4.89 | -34 | 13 | 20 | Left inferior frontal gyrus p ope |
|  |  | 4.68 | -36 | 18 | 30 | Left middle frontal gyrus |
|  |  | 4.56 | -46 | 28 | 22 | Left inferior frontal gyrus p tri |
|  |  | 4.31 | -46 | 33 | 7 | Left inferior frontal gyrus p tri |
|  |  | 4.17 | -54 | 23 | 30 | Left middle frontal gyrus |
|  |  | 4.06 | -44 | 26 | 10 | Left inferior frontal gyrus p tri |
|  |  | 3.29 | -56 | 33 | 14 | Left inferior frontal gyrus p tri |
|  | 97 | 4.87 | -31 | -74 | 47 | Left lateral occipital cortex sup |
|  | 613 | 4.79 | -24 | -94 | 10 | Left occipital pole |
|  |  | 4.56 | -21 | -94 | 20 | Left occipital pole |
|  |  | 3.83 | -9 | -90 | 17 | Left occipital pole |
|  |  | 3.81 | -21 | -90 | 32 | Left occipital pole |
|  | 109 | 4.74 | 31 | 36 | -10 | Right frontal pole |
|  |  | 4.07 | 14 | 30 | -10 | right uncinate fas |
|  | 80 | 4.36 | 14 | -82 | -46 | Right cerebellum |
|  |  | 3.93 | 31 | -70 | -43 | Right cerebellum |
|  |  | 3.48 | 36 | -77 | -46 | Right cerebellum |
| ME > SE | 1309 | 6.42 | -44 | -40 | -18 | Left temporal fusiform cortex pos |
|  |  | 5.93 | -51 | -44 | -13 | left superior longitudinal fas |
|  |  | 5.69 | -41 | -47 | -8 | left inferior longitudinal fas |
|  |  | 5.14 | -54 | -57 | -20 | Left inferior temporal gyrus temocc |
|  |  | 5.01 | -46 | -52 | -20 | Left inferior temporal gyrus temocc |
|  |  | 4.9 | -56 | -77 | 14 | Left lateral occipital cortex inf |
|  |  | 4.71 | -46 | -42 | 10 | Left superior temporal gyurs pos |
|  |  | 4.58 | -39 | -24 | -20 | Left temporal fusiform cortex pos |
|  | 348 | 6.13 | 36 | -40 | -28 | Right temporal fusiform cortex pos |
|  |  | 4.35 | 24 | -52 | -18 | Right temporal occipital fusiform cortex |
|  |  | 4.19 | 34 | -22 | -36 | Right temporal fusiform cortex pos |
|  |  | 3.98 | 44 | -32 | -23 | Right inferior temporal gyrus post |
|  |  | 3.9 | 34 | -12 | -33 | Right temporal fusiform cortex pos |
|  |  | 3.73 | 46 | -44 | -16 | Right inferior temporal gyrus temocc |
|  |  | 3.27 | 24 | -44 | -23 | Right temporal occipital fusiform cortex |
|  | 562 | 5.51 | -19 | 36 | -13 | Left frontal orbital cortex |
|  |  | 5.12 | 31 | 38 | -10 | Right frontal pole |
|  |  | 4.84 | 9 | 8 | -16 | Right subcallosal cortex |
|  |  | 4.66 | 14 | 30 | -10 | right uncinate fas |
|  |  | 4.44 | -9 | 23 | -13 | Left subcallosal cortex |
|  |  | 3.89 | -34 | 36 | -10 | Left frontal orbital cortex |
|  |  | 3.77 | 1 | 18 | -18 | Right subcallosal cortex |
|  |  | 3.76 | 11 | 20 | -13 | Right subcallosal cortex |
|  | 134 | 4.95 | 14 | -82 | -46 | Right cerebellum |
|  |  | 3.64 | 26 | -77 | -46 | Right cerebellum |
|  |  | 3.53 | 11 | -82 | -36 | Right cerebellum |
|  |  | 3.4 | 14 | -72 | -36 | Right cerebellum |
|  | 92 | 4.34 | 54 | -74 | -10 | Right lateral occipital cortex inf |
|  |  | 4.32 | 49 | -84 | 2 | Right lateral occipital cortex inf |
|  |  | 4.11 | 54 | -77 | 10 | Right lateral occipital cortex inf |
| SE > ME | 93 | 4.89 | -36 | 10 | 17 | Left frontal operculum cortex |
|  |  | 3.71 | -34 | -2 | 32 | Left precentral gyrus |
| MB > SB | 3959 | 6.7 | -44 | -77 | 2 | Left lateral occipital cortex inf |
|  |  | 6.58 | -49 | -77 | 10 | Left lateral occipital cortex inf |
|  |  | 6.53 | -34 | -47 | -20 | Left temporal occipital fusiform cortex |
|  |  | 6.43 | -36 | -72 | -16 | Left occipital fusiform gyrus |
|  |  | 6.28 | -41 | -80 | -10 | Left lateral occipital cortex inf |
|  |  | 6.26 | -36 | -54 | -18 | Left temporal occipital fusiform cortex |
|  |  | 5.95 | -44 | -50 | -16 | Left inferior temporal gyrus temocc |
|  |  | 5.7 | -29 | -54 | -13 | Left temporal occipital fusiform cortex |
|  | 2569 | 6.18 | 46 | -67 | 12 | Right lateral occipital cortex inf |
|  |  | 6.1 | 46 | -77 | -3 | Right lateral occipital cortex inf |
|  |  | 6.01 | 36 | -74 | -13 | Right occipital fusiform gyrus |
|  |  | 5.65 | 31 | -62 | -16 | Right occipital fusiform gyrus |
|  |  | 5.62 | 46 | -77 | 7 | Right lateral occipital cortex inf |
|  |  | 5.49 | 29 | -90 | 14 | Right occipital pole |
|  |  | 5.35 | 36 | -52 | -20 | Right temporal occipital fusiform cortex |
|  |  | 5.3 | 34 | -34 | -23 | Right temporal fusiform cortex pos |
|  | 828 | 4.68 | -54 | 23 | 30 | Left middle frontal gyrus |
|  |  | 4.62 | -46 | 33 | 12 | Left inferior frontal gyrus p tri |
|  |  | 4.47 | -46 | 26 | 22 | Left inferior frontal gyrus p tri |
|  |  | 4.39 | -39 | 16 | 30 | Left middle frontal gyrus |
|  |  | 4.12 | -51 | 20 | 42 | Left middle frontal gyrus |
|  |  | 3.59 | -44 | 38 | -6 | Left frontal pole |
|  |  | 3.51 | -46 | 48 | 7 | Left frontal pole |
|  |  | 3.44 | -39 | 23 | 52 | Left middle frontal gyrus |
| SB > MB | 427 | 4.36 | 44 | -44 | 57 | Right superior parietal lobule |
|  |  | 4.2 | 46 | -40 | 42 | Right supramarginal gyrus pos |
|  |  | 3.65 | 56 | -32 | 50 | Right supramarginal gyrus ant |
|  |  | 3.64 | 31 | -47 | 64 | Right superior parietal lobule |
|  |  | 3.53 | 59 | -40 | 52 | Right supramarginal gyrus pos |
|  |  | 3.49 | 26 | -44 | 57 | Right superior parietal lobule |
|  |  | 3.35 | 61 | -47 | 37 | Right supramarginal gyrus pos |
|  | 130 | 3.95 | -4 | -60 | 64 | Left precuneous cortex |
|  |  | 3.59 | -11 | -57 | 74 | Left superior parietal lobule |
|  |  | 3.56 | 1 | -52 | 67 | Right precuneous cortex |
|  |  | 3.47 | -21 | -57 | 62 | Left superior parietal lobule |
|  |  | 3.45 | -4 | -54 | 54 | Left precuneous cortex |
|  |  | 3.37 | -11 | -64 | 62 | Left lateral occipital cortex sup |
| 2 x 2 factorial design – denoising and band | | | | | | |
| ANOVA | 6202 | Inf | 36 | -32 | -26 | Right temporal fusiform cortex pos |
|  |  | 6.88 | 39 | -40 | -20 | Right temporal occipital fusiform cortex |
|  |  | 6.64 | 26 | -37 | -23 | Right temporal fusiform cortex pos |
|  |  | 6.51 | -36 | -44 | -16 | Left temporal occipital fusiform cortex |
|  |  | 6.45 | -39 | -34 | -28 | Left temporal fusiform cortex pos |
|  |  | 6.28 | -44 | -37 | -20 | Left inferior temporal gyrus post |
|  |  | 6.15 | 41 | -77 | -18 | Right lateral occipital cortex inf |
|  |  | 6.03 | -39 | -20 | -26 | Left temporal fusiform cortex pos |
|  | 630 | 4.77 | -44 | 30 | 4 | Left inferior frontal gyrus p tri |
|  |  | 4.68 | -39 | 13 | 22 | Left inferior frontal gyrus p ope |
|  |  | 4.56 | -56 | 40 | 2 | Left frontal pole |
|  |  | 4.56 | -44 | 23 | 14 | Left inferior frontal gyrus p tri |
|  |  | 4.32 | -36 | 40 | -6 | Left frontal pole |
|  |  | 4.3 | -56 | 30 | -3 | Left inferior frontal gyrus p tri |
|  |  | 4.12 | -39 | 13 | 34 | Left middle frontal gyrus |
|  |  | 3.92 | -54 | 38 | 17 | Left frontal pole |
|  | 286 | 4.59 | 14 | -97 | 27 | Right occipital pole |
|  |  | 4.51 | 21 | -94 | 20 | Right occipital pole |
|  |  | 4.35 | 11 | -90 | 22 | Right occipital pole |
|  |  | 4 | 9 | -92 | 32 | Right occipital pole |
|  |  | 3.95 | 31 | -100 | 12 | Right occipital pole |
|  | 118 | 4.55 | -16 | -97 | 0 | Left occipital pole |
|  |  | 3.96 | -26 | -92 | 10 | Left occipital pole |
|  | 413 | 4.42 | 44 | -40 | 42 | Right supramarginal gyrus pos |
|  |  | 4.28 | 54 | -37 | 42 | Right supramarginal gyrus pos |
|  |  | 4.23 | 49 | -37 | 32 | right superior longitudinal fas |
|  |  | 3.98 | 44 | -42 | 52 | Right supramarginal gyrus pos |
|  |  | 3.86 | 29 | -47 | 42 | Right superior parietal lobule |
|  |  | 3.61 | 61 | -47 | 44 | Right angular gyrus |
|  |  | 3.5 | 59 | -37 | 34 | Right supramarginal gyrus pos |
|  | 91 | 4.39 | -41 | 36 | -20 | Left frontal pole |
|  | 93 | 4.17 | 64 | -24 | 20 | Right parietal operculum cortex |
|  |  | 3.99 | 66 | -27 | 27 | Right supramarginal gyrus ant |
|  | 196 | 4.08 | -16 | -90 | 27 | Left occipital pole |
|  |  | 4.03 | -1 | -94 | 22 | Left occipital pole |
|  |  | 3.97 | -4 | -87 | 24 | Left cuneal cortex |
|  |  | 3.91 | -26 | -94 | 30 | Left occipital pole |
|  |  | 3.88 | -29 | -87 | 27 | Left lateral occipital cortex sup |
|  | 85 | 3.94 | -1 | -50 | 52 | Left precuneous cortex |
|  |  | 3.7 | 11 | -60 | 54 | Right precuneous cortex |
|  |  | 3.26 | -4 | -62 | 57 | Left precuneous cortex |
| MEdn > ME | 8383 | 7.46 | 36 | -32 | -23 | Right temporal fusiform cortex pos |
|  |  | 7.22 | 26 | -37 | -23 | Right temporal fusiform cortex pos |
|  |  | 6.76 | 34 | -40 | -26 | Right temporal occipital fusiform cortex |
|  |  | 6.61 | 41 | -77 | -18 | Right lateral occipital cortex inf |
|  |  | 6.54 | -39 | -37 | -26 | Left temporal fusiform cortex pos |
|  |  | 6.53 | -36 | -44 | -16 | Left temporal occipital fusiform cortex |
|  |  | 6.51 | 21 | -47 | -18 | Right temporal occipital fusiform cortex |
|  |  | 6.45 | 29 | -52 | -16 | Right temporal occipital fusiform cortex |
|  | 263 | 4.93 | 14 | -97 | 27 | Right occipital pole |
|  |  | 4.37 | 4 | -92 | 32 | Right occipital pole |
|  |  | 4.32 | -1 | -94 | 24 | Left occipital pole |
|  |  | 4.22 | 31 | -100 | 12 | Right occipital pole |
|  |  | 4.03 | -19 | -100 | 27 | Left occipital pole |
|  |  | 3.78 | 26 | -97 | 22 | Right occipital pole |
|  |  | 3.75 | -19 | -92 | 34 | Left occipital pole |
|  |  | 3.6 | -4 | -87 | 40 | Left cuneal cortex |
|  | 102 | 4.92 | -11 | 18 | 50 | Left superior frontal gyrus |
|  |  | 3.64 | -6 | 20 | 42 | Left paracingulate gyrus |
|  |  | 3.56 | -1 | 23 | 50 | Left superior frontal gyrus |
|  | 129 | 4.74 | 39 | 23 | 14 | Right inferior frontal gyrus p tri |
|  |  | 3.71 | 59 | 30 | 10 | Right inferior frontal gyrus p tri |
|  |  | 3.67 | 56 | 28 | 20 | Right inferior frontal gyrus p tri |
|  | 88 | 4.25 | 29 | -37 | 14 | Right lateral ventricle |
|  |  | 3.98 | 21 | -27 | 20 | Right lateral ventricle |
|  |  | 3.48 | 26 | -32 | 7 | Right lateral ventricle |
|  |  | 3.4 | 34 | -44 | 10 | right inferior frontal occipital fas |
| ME > MEdn | 80 | 4.54 | -71 | -20 | 2 | Left superior temporal gyurs pos |
|  |  | 3.76 | -61 | -24 | 7 | Left planum temporale |
|  |  | 3.23 | -71 | -30 | 7 | Left superior temporal gyurs pos |
|  | 112 | 4.42 | 1 | -14 | 27 | Right cingulate gyrus ant |
|  |  | 3.73 | 9 | -24 | 34 | right cingulum cingulate |
|  |  | 3.69 | 6 | -22 | 24 | Right lateral ventricle |
|  | 163 | 4.22 | -1 | 30 | 14 | Left cingulate gyrus ant |
|  |  | 4.09 | 1 | 38 | -6 | Right cingulate gyrus ant |
|  |  | 3.81 | -4 | 36 | 7 | Left cingulate gyrus ant |
|  |  | 3.65 | 6 | 46 | 0 | Right paracingulate gyrus |
|  |  | 3.36 | 16 | 48 | 2 | forceps minor |
|  | 134 | 4.03 | 21 | -42 | -30 | Right corticospinal tract |
|  |  | 4.01 | 16 | -57 | -48 | Right cerebellum |
|  |  | 3.59 | 14 | -47 | -26 | Right cerebellum |
|  |  | 3.4 | 19 | -52 | -38 | Right corticospinal tract |
| 2 x 2 factorial design – echo and (downsampled) band | | | | | | |
| ANOVA | 486 | 5.4 | -44 | -40 | -18 | Left temporal fusiform cortex pos |
|  |  | 4.85 | -41 | -47 | -10 | left inferior longitudinal fas |
|  |  | 4.79 | -51 | -44 | -13 | left superior longitudinal fas |
|  |  | 4.58 | -46 | -52 | -20 | Left inferior temporal gyrus temocc |
|  |  | 4.44 | -34 | -44 | -20 | Left temporal fusiform cortex pos |
|  |  | 4.16 | -39 | -24 | -20 | Left temporal fusiform cortex pos |
|  |  | 4.02 | -36 | -52 | -18 | Left temporal occipital fusiform cortex |
|  |  | 3.82 | -59 | -40 | -10 | Left middle temporal gyrus pos |
|  | 281 | 5.39 | 44 | -34 | -23 | Right inferior temporal gyrus post |
|  |  | 5.19 | 36 | -37 | -28 | Right temporal fusiform cortex pos |
|  |  | 3.77 | 49 | -47 | -16 | Right inferior temporal gyrus temocc |
|  |  | 3.74 | 39 | -24 | -18 | Right temporal fusiform cortex pos |
|  |  | 3.7 | 34 | -40 | -18 | Right temporal fusiform cortex pos |
|  |  | 3.28 | 36 | -54 | -20 | Right temporal occipital fusiform cortex |
|  |  | 3.21 | 26 | -42 | -16 | Right temporal occipital fusiform cortex |
|  | 78 | 4.63 | -19 | 36 | -13 | Left frontal orbital cortex |
|  |  | 3.5 | -19 | 28 | -18 | Left frontal orbital cortex |
|  |  | 3.35 | -9 | 23 | -13 | Left subcallosal cortex |
|  | 57 | 4.39 | 31 | 36 | -10 | Right frontal pole |
|  |  | 3.22 | 21 | 38 | -13 | Right frontal pole |
|  | 116 | 4.37 | -36 | 18 | 30 | Left middle frontal gyrus |
|  |  | 4.13 | -36 | 13 | 17 | Left frontal operculum cortex |
|  |  | 3.88 | -34 | 20 | 20 | Left inferior frontal gyrus p ope |
|  |  | 3.83 | -46 | 26 | 24 | Left middle frontal gyrus |
|  | 64 | 4.19 | 41 | -70 | 10 | Right lateral occipital cortex inf |
|  |  | 4.01 | 44 | -60 | 12 | Right lateral occipital cortex inf |

Supplementary table 2: Significant cluster and peak information for main effects, ANOVA effects of interest and directed effects of interest when comparing activation magnitude and activation precision. All coordinates are given in MNI space and all anatomical labels are extracted from the Harvard-Oxford cortical and subcortical structural atlases.

Supplementary figure 1: Effects of parallel transmit on activation magnitude. (A) effects of the SESB sequence and the pTx sequence for the contrast of interest (S > C); (B) effect of parallel transmit (pTx > SESB, red; SESB > pTx, no significant clusters). Results are cluster-corrected at p < 0.05 based on an uncorrected voxel threshold of p < 0.001 and are overlaid on the MNI152NLin2009cAsym template.

Supplementary figure 2: Effects of parallel transmit on activation precision. Effects of the SESB sequence and the pTx sequence for the contrast of interest (S > C). No contrasts (pTx > SESB, SESB > pTx) were significant. Results are cluster-corrected at p < 0.05 based on an uncorrected voxel threshold of p < 0.001 and are overlaid on the MNI152NLin2009cAsym template.

Supplementary figure 3: Effects of multi-echo and multiband on activation magnitude. (A) main effect of each sequence for the contrast of interest (S > C); (B) effect of echo (ME > SE, red; SE > ME, no significant clusters). No effects of multiband (MB > SB; SB > MB) were significant. Results are cluster-corrected at p < 0.05 based on an uncorrected voxel threshold of p < 0.001 and are overlaid on the MNI152NLin2009cAsym template.

Supplementary figure 4: Effects of multi-echo and multiband on activation precision. (A) main effect of each sequence (S > C); (B) effect of echo (ME > SE, red; SE > ME, blue); (C) effect of band (MB > SB, red; SB > MB, blue). Results are cluster-corrected at p < 0.05 based on an uncorrected voxel threshold of p < 0.001 and are overlaid on the MNI152NLin2009cAsym template.

Supplementary figure 5: Effect of ME-ICA denoising on activation magnitude (MEdn > ME, red; ME > MEdn, no significant clusters). Results are cluster-corrected at p < 0.05 based on an uncorrected voxel threshold of p < 0.001 and are overlaid on the MNI152NLin2009cAsym template.

Supplementary figure 6: Effect of ME-ICA denoising on activation precision (MEdn > ME, red; ME > MEdn, blue). Results are cluster-corrected at p < 0.05 based on an uncorrected voxel threshold of p < 0.001 and are overlaid on the MNI152NLin2009cAsym template.

Supplementary figure 7: Slice leakage analysis (all peaks). (A) Seed and possible artefact locations for a single participant. The ROIs (radius 4 voxels) indicate the seed location (blue), possible artefact location based on phase shift (green), possible artefact location based on GRAPPA (red), and possible artefact location based on phase shift and GRAPPA (yellow) in native EPI space. Informed consent was obtained from the participant for this image to be published. (B) Mean activation magnitude within each sphere for each MB sequence and the corresponding control sequence for each seed location. (C) Mean MVPA dissimilarity within each sphere for each MB sequence and the corresponding control sequence.

|  | Peak 1 | Peak 2 | Peak 3 | Peak 4 | Peak 5 | |
| --- | --- | --- | --- | --- | --- | --- |
| Activation magnitude | | | | | |  |
| SEMB_B_ | 0.3228 | 0.8153 | 0.3865 | 0.2161 | 0.6244 | |
| MEMB_Ag_ | 0.0539 | 0.9614 | 0.3099 | 0.0068* | 0.5378 | |
| MEMB_B_ | 0.2499 | 0.5327 | 0.3019 | 0.7382 | 0.5362 | |
| MEMB_Bg_ | 0.9647 | 0.3988 | 0.8770 | 0.9917 | 0.5296 | |
| MVPA | | | | | |  |
| SEMB_B_ | 0.0596 | 0.3208 | 0.0107* | 0.1079 | 0.3597 | |
| MEMB_Ag_ | 0.0974 | 0.1860 | 0.2596 | 0.0959 | 0.3434 | |
| MEMB_B_ | 0.2528 | 0.6570 | 0.0354* | 0.0083* | 0.8442 | |
| MEMB_Bg_ | 0.9408 | 0.8805 | 0.5601 | 0.8335 | 0.5945 | |

Supplementary table 3: p-values for all slice leakage tests. A is the seed location, B is the possible artefact location based on phase shift, Ag is the possible artefact location in the same slice as A based on GRAPPA, and Bg is the possible artefact location in the same slice as B based on GRAPPA. * = p < 0.05, ** = p < 0.05 (Bonferroni-corrected for the number of peaks and possible artefact regions), SEMB = single-echo multiband, multiband, MEMB = multi-echo multiband.

**Supplementary Material 2: Investigating the impact of Ernst angle on the effects of interest**

1. **Introduction**

Due to experimenter error, the flip angle for our SESB and SEMB was not set to the Ernst angle (SESB: nominal flip angle = 50° compared to Ernst angle of 78°; SEMB: nominal flip angle of 50° compared to Ernst angle of 63°). Theoretically, use of suboptimal flip angles would result in a tSNR loss relative to using the Ernst angle – 12.99% for the SESB sequence and 3.15% for the SEMB sequence – and this loss might explain the poorer performance of SE sequences relative to ME sequences. However, because of B_1_ inhomogeneity at 7T, the effective flip angle may differ from the nominal flip angle and so the actual signal loss may differ from theoretical predictions. This difference may vary across the brain, especially for CP-mode scans (Uğurbil, 2018), so it is important to determine the actual effect within ventral temporal and orbitofrontal regions. We acquired data to test the impact of suboptimal flip angle on tSNR. Since tSNR is known to dissociate from functional contrast under some circumstances (Ding et al., 2022), we also tested the impact on our effects of interest (activation magnitude, activation precision, and our MVPA metric) within our ROIs.

2. **Methods**

We collected data from two healthy participants who did not participate in the main study and who gave informed consent. There were four functional runs per participant, collected in a single session. The first two runs used the SE sequences described in the main text (Run 1 – SESB, Run 2 – SEMB). The other two runs used SE sequences in which the nominal flip angle was set to the Ernst angle (all other parameters were kept constant between matched sequences; Run 3 – SESBernst, Run 4 - SEMBernst). All other aspects of the methods, including task, preprocessing, and GLMs, were identical to those described in the main text. tSNR, activation magnitude, activation precision, and MVPA performance were calculated within every ROI reported in the main text.

3. **Results**

Supplementary table 4 shows the results. Values in the table are percentage improvement using the optimised sequence relative to the sequence reported in the main text. In both participants, use of the Ernst angle conferred a tSNR increase that was relatively consistent across ROIs. The difference between the suboptimal flip angle and the Ernst angle was greater for the SESB sequence than for the SEMB sequence; consistent with this, the improvement in tSNR when using the optimised sequence was greater for the SESB sequence. These tSNR results were therefore in line with what would be expected from a sequence using the Ernst angle. However, our three effects of interest – activation magnitude, activation precision, and our MVPA metric – showed an inconsistent pattern. The magnitude and direction of change when using the optimised sequence relative to the suboptimal sequence varied across ROIs and across participants.

|  | LTP | LvATL | RITG | LFP | LmMTG | LpMTG | LIFGpt |
| --- | --- | --- | --- | --- | --- | --- | --- |
| **tSNR** | | | | | | | |
| Participant 1 | | | | | | | |
| SESBernst > SESB | 54.0286 | -9.9851 | 59.8135 | 19.7037 | 14.1672 | 4.3193 | 19.8550 |
| SEMBernst > SEMB | 34.4127 | -20.3186 | 3.6741 | 12.7745 | 3.0920 | 3.2001 | 13.1559 |
| Participant 2 | | | | | | | |
| SESBernst > SESB | -3.2123 | 19.6513 | 87.5284 | 52.9209 | 20.7838 | 24.4694 | -5.9168 |
| SEMBernst > SEMB | 24.7779 | -0.7088 | -7.1845 | 31.0988 | 0.3452 | 9.3827 | 1.0219 |
| **Activation magnitude** | | | | | | | |
| Participant 1 | | | | | | | |
| SESBernst > SESB | 44.0782 | 583.4712 | 227.3575 | -742.3157 | -211.2123 | 46.1007 | 1564.3744 |
| SEMBernst > SEMB | -3.2425 | -45.0988 | -85.7035 | -426.4440 | -36.8633 | -91.4717 | -99.7580 |
| Participant 2 | | | | | | | |
| SESBernst > SESB | -57.6951 | 11.2470 | -1.9989 | -75.8461 | -147.4325 | -55.1979 | -102.6642 |
| SEMBernst > SEMB | -36.7422 | -39.1730 | -16.8902 | 191.7208 | 82.8526 | -57.2460 | -7.2006 |
| **Activation precision** | | | | | | | |
| Participant 1 | | | | | | | |
| SESBernst > SESB | 63.3282 | 588.9795 | 219.5805 | -1345.4358 | -220.6662 | 73.0525 | 1588.8752 |
| SEMBernst > SEMB | -0.4183 | -37.5597 | -88.0863 | -412.9069 | -20.8991 | -88.6187 | -100.0000 |
| Participant 2 | | | | | | | |
| SESBernst > SESB | -65.4802 | 19.5963 | -7.2510 | -75.8659 | -155.2239 | -60.3942 | -101.5743 |
| SEMBernst > SEMB | -34.6221 | -41.4298 | 16.2403 | 133.2690 | 83.1263 | -52.9855 | -9.2875 |
| **MVPA** | | | | | | | |
| Participant 1 | |  |  |  |  |  |  |
| SESBernst > SESB | -23.9196 | 122.1070 | 47.6859 | 154.8006 | -32.3599 | 35.5384 | 35.1941 |
| SEMBernst > SEMB | 63.3551 | -111.4677 | -149.0596 | 50.2805 | -1.8231 | -297.4278 | -120.5662 |
| Participant 2 | | | | | | | |
| SESBernst > SESB | -107.5576 | -24.3165 | -109.6968 | -85.2973 | 282.0658 | -129.8579 | -128.2633 |
| SEMBernst > SEMB | -211.9854 | -89.6384 | 49.8018 | -19172.9623 | 5.6581 | -2058.3843 | -78.0659 |

Supplementary table 4: Impact of suboptimal flip angles on tSNR and effects of interest. Values are percentage improvement using the optimised sequence relative to the sequence reported in the main text. SESB = single-echo single band sequence reported in main text, SESBernst = single-echo single band sequence using Ernst angle as nominal flip angle, SEMB = single-echo multiband sequence reported in main text, SEMBernst = single-echo multiband sequence using Ernst angle as nominal flip angle, LTP = left temporal pole, LvATL = left ventral anterior temporal lobe, RITG = right inferior temporal gyrus, LFP = left frontal pole, LmMTG = left medial middle temporal gyrus, LpMTG = left posterior middle temporal gyrus, LIFGpt = left inferior temporal gyrus pars triangularis.

4. **Discussion**

To thoroughly eliminate all possible confounds, future studies investigating the effect of multi-echo sequences relative to single-echo sequences should strive to set the flip angle to the Ernst angle and to ensure that this is consistent across sequences. However, these analyses suggest that suboptimal flip angle alone cannot explain the benefits of ME over SE for our outcome effects of interest (activation magnitude, activation precision, and MVPA performance) that was observed in the main study. It is possible that a group difference would emerge in a well-powered study designed specifically to investigate the effect of flip angle; however, these preliminary results are sufficient to highlight large interindividual variability. These results also align with previous work that finds a dissociation between tSNR and functional contrast metrics (Ding et al., 2022).

We therefore conclude that the difference in flip angle is unlikely to explain the reported advantage of ME over SE sequences.
